# Establishment and activity of the planning and acting network for low dose radiation research in Japan (PLANET): 2016–2023

**DOI:** 10.1093/jrr/rrae049

**Published:** 2024-07-15

**Authors:** Yutaka Yamada, Tatsuhiko Imaoka, Toshiyasu Iwasaki, Junya Kobayashi, Munechika Misumi, Kazuo Sakai, Takashi Sugihara, Keiji Suzuki, Hiroshi Tauchi, Hiroshi Yasuda, Shinji Yoshinaga, Megumi Sasatani, Satoshi Tanaka, Kazutaka Doi, Masanori Tomita, Daisuke Iizuka, Shizuko Kakinuma, Michiya Sasaki, Michiaki Kai

**Affiliations:** Department of Radiation Effects Research, Institute for Radiological Science, National Institutes for Quantum Science and Technology, 4-9-1 Anagawa, Inage-ku, Chiba 263-8555, Japan; Department of Radiation Effects Research, Institute for Radiological Science, National Institutes for Quantum Science and Technology, 4-9-1 Anagawa, Inage-ku, Chiba 263-8555, Japan; Sustainable System Research Laboratory, Central Research Institute of Electric Power Industry, 1646 Abiko, Abiko, Chiba 270-1194, Japan; Department of Radiological Sciences, School of Health Sciences at Narita, International University of Health and Welfare, 4-3, Kozunomori, Narita, Chiba 286-8686, Japan; Department of Statistics, Radiation Effects Research Foundation, 5-2 Hijiyama Park, Minami-ku, Hiroshima 732-0815, Japan; Tokyo Healthcare University, 2-5-1 Higashiaoka, Meguro-ku, Tokyo 152-8558, Japan; Department of Radiobiology, Institute for Environmental Sciences, 1-7 Ienomae, Obuchi, Rokkasho-mura, Kamikita-gun, Aomori 039-3212, Japan; Department of Radiation Medical Sciences, Atomic Bomb Disease Institute, Nagasaki University, 1-12-4 Sakamoto, Nagasaki 852-8523, Japan; Department of Biological Sciences, Ibaraki University, Bunkyo 2-1-1, Mito, Ibaraki 310-8512, Japan; Department of Radiation Biophysics, Research Institute for Radiation Biology and Medicine, Hiroshima University, 1-2-3 Kasumi, Minami-ku, Hiroshima 734-8553, Japan; Department of Environmetrics and Biometrics, Research Institute for Radiation Biology and Medicine, Hiroshima University, 1-2-3 Kasumi, Minami-ku, Hiroshima 734-8553, Japan; Department of Experimental Oncology, Research Institute for Radiation Biology and Medicine, Hiroshima University, 1-2-3 Kasumi, Minami-ku, Hiroshima 734-8553, Japan; Department of Radiobiology, Institute for Environmental Sciences, 1-7 Ienomae, Obuchi, Rokkasho-mura, Kamikita-gun, Aomori 039-3212, Japan; Department of Radiation Regulatory Science Research, Institute for Radiological Science, National Institutes for Quantum Science and Technology, 4-9-1 Anagawa, Inage-ku, Chiba 263-8555, Japan; Sustainable System Research Laboratory, Central Research Institute of Electric Power Industry, 1646 Abiko, Abiko, Chiba 270-1194, Japan; Department of Radiation Effects Research, Institute for Radiological Science, National Institutes for Quantum Science and Technology, 4-9-1 Anagawa, Inage-ku, Chiba 263-8555, Japan; Department of Radiation Effects Research, Institute for Radiological Science, National Institutes for Quantum Science and Technology, 4-9-1 Anagawa, Inage-ku, Chiba 263-8555, Japan; Sustainable System Research Laboratory, Central Research Institute of Electric Power Industry, 1646 Abiko, Abiko, Chiba 270-1194, Japan; Nippon Bunri University, 1727-162 Ichiki, Oita, Oita 870-0397, Japan

**Keywords:** radiation, low dose, low dose rate, biology, modeling

## Abstract

The Planning and Acting Network for Low Dose Radiation Research in Japan (PLANET) was established in 2017 in response to the need for an all-Japan network of experts. It serves as an academic platform to propose strategies and facilitate collaboration to improve quantitative estimation of health risks from ionizing radiation at low-doses and low-dose-rates. PLANET established Working Group 1 (Dose-Rate Effects in Animal Experiments) to consolidate findings from animal experiments on dose-rate effects in carcinogenesis. Considering international trends in this field as well as the situation in Japan, PLANET updated its priority research areas for Japanese low-dose radiation research in 2023 to include (i) characterization of low-dose and low-dose-rate radiation risk, (ii) factors to be considered for individualization of radiation risk, (iii) biological mechanisms of low-dose and low-dose-rate radiation effects and (iv) integration of epidemiology and biology. In this context, PLANET established Working Group 2 (Dose and Dose-Rate Mapping for Radiation Risk Studies) to identify the range of doses and dose rates at which observable effects on different endpoints have been reported; Working Group 3 (Species- and Organ-Specific Dose-Rate Effects) to consider the relevance of stem cell dynamics in radiation carcinogenesis of different species and organs; and Working Group 4 (Research Mapping for Radiation-Related Carcinogenesis) to sort out relevant studies, including those on non-mutagenic effects, and to identify priority research areas. These PLANET activities will be used to improve the risk assessment and to contribute to the revision of the next main recommendations of the International Commission on Radiological Protection.

## INTRODUCTION

The current system of radiological protection focuses on the health risks of low-dose and low-dose-rate radiation exposure. The United Nations Scientific Committee on the Effects of Atomic Radiation (UNSCEAR) defines low-dose and low-dose-rate for the purpose of radiation protection as <100 mGy and < 0.1 mGy/min (i.e. <6 mGy/h), respectively [1]. However, the underlying risk assessment is primarily based on epidemiological studies of Japanese atomic bomb survivors exposed to acute high-dose radiation. It has been proposed that, for low-dose and low-dose-rate radiation exposures, a dose and dose-rate effectiveness factor (DREF) should be applied irrespective of the total dose received to reduce excess cancer risk estimates based on evidence from high-dose acute exposures [2]. It is difficult to quantitatively assess the risk of low-dose and low-dose-rate radiation exposure because of statistical limitations in detecting risk at several tens of mGy or less and the uncertainties of epidemiological studies on populations receiving low-dose-rate radiation. In Japan, the accident at the Tokyo Electric Power Company Fukushima Daiichi Nuclear Power Plant and the increasing use of various types of radiation in medicine have made reasonable risk assessment of low-dose and low-dose-rate radiation exposure increasingly necessary, as it is crucial for radiation regulation and related social consensus building. One possible measure to address this issue is to establish an all-Japan network of academic and research institutions to promote collaboration among relevant parties, including regulatory authorities, and to establish and implement a research agenda for low-dose and low-dose-rate radiation. The Planning and Acting Network for Low Dose Radiation Research in Japan (PLANET) was thus established in 2017.

The present article summarizes PLANET activity by presenting PLANET’s prioritization of important research areas related to risk estimation of and protection from low-dose and low-dose-rate radiation in Japan and its efforts to evaluate the current state of research in various related fields. An important goal of this work is to inform and encourage discussion for the International Commission on Radiological Protection (ICRP)’s upcoming revision of its 2007 recommendations [3].

## ESTABLISHMENT AND ACTIVITY OF PLANET, 2016–2023

### Establishment of PLANET

In 2016, the National Institutes for Quantum Science and Technology set up a preparatory committee for the above-mentioned nationwide network and compiled a report in March 2017 [4]. The network was named PLANET and was expected to (i) prioritize research needs taking into account achievements at research institutes and universities conducting research on low-dose radiation effects in Japan, (ii) propose strategies to improve the estimation of low-dose and low-dose-rate radiation risks, (iii) propose a support system for cooperation and collaboration with relevant domestic researchers and research institutions and (iv) promote partnerships with international organizations.

**Fig. 1:**
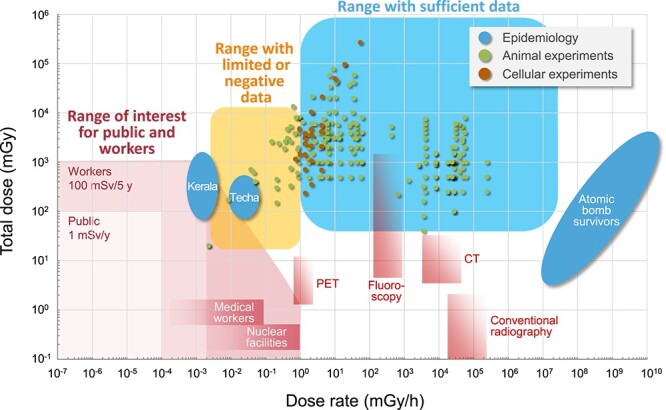
Doses and dose rates at which data are available from major animal and cellular experiments and epidemiological studies (adapted from PLANET Preparatory Committee [4]). Animal and cellular experiments are those containing conditions <6 mGy/h in dose rate and > 2 weeks in exposure duration and animal studies reported by Haley *et al*. [10]. CT, X-ray computed tomography; PET, positron emission tomography.

Figure 1 shows the dose and dose-rate ranges for which experimental and epidemiological data existed at the time and the ranges relevant to protection of the public and workers. The graph shows the paucity of data at low-dose-rate below ~1 mGy/h; in addition, there were little data, either epidemiological or biological, within the range of interest for protection of the public and workers. The risk estimation for radiological protection has thus been based mainly on epidemiological data from atomic bomb survivors at high-dose-rates, with reference to other data that existed mainly in the data-rich range above 1 mGy/h.

In Japan, major societal concerns related to radiation include the existing exposure of the public after the Fukushima accident, occupational exposure of radiation workers (including cleanup workers following the accident) and increasing medical exposure from widely used and advanced techniques. Epidemiological cohort studies on emergency workers at Fukushima Daiichi (the Nuclear Energy Workers Study or NEWS) and on Japanese nuclear workers (the Japanese Epidemiological Study on Low-Dose Radiation Effects or J-EPISODE) are underway. Nevertheless, in epidemiological data alone, it is difficult to quantitatively assess risk at low-doses because of statistical limitations and possible insufficient adjustment for confounding factors. One way to reduce uncertainty in risk estimation of low-dose and low-dose-rate radiation is through the integration of biology and epidemiology. For the integration, a key issue is how to quantitatively model the effects of doses and dose rates that are relevant to radiological protection. To achieve this, it is important to accumulate empirical data through both epidemiological and experimental approaches and integrate them for use in risk estimation using mathematical models. It is also essential to identify biomarkers that represent early radiation-induced events leading to disease and integrate the findings with those of epidemiological and animal experiments.

To this end, the PLANET Preparatory Committee recommended the following five categories as prioritized research areas:

(1) Epidemiological studies of low-dose and low-dose-rate radiation appropriately designed for risk estimation
(2) Mechanistic elucidation of low-dose and low-dose-rate radiation effects for risk estimation
(3) Integration of biological data for interpretation of epidemiological studies
(4) Association of age, sex, genetic predisposition and lifestyle with radiation risk
(5) Data collection and database compilation/archiving including negative data

In response, the PLANET Steering Committee was established in 2017 to address these priority issues.

### Establishment and activity of Working Group 1: Dose-rate effects in animal models

Epidemiology has contributed significantly to estimation of the dose and DREF, which was discussed separately as a low-dose extrapolation factor and a DREF [5–8]. Studies using animal models have contributed substantially to providing qualitative and quantitative evidence, including evidence for relevant biological mechanisms [7–10]. Because PLANET viewed that the consolidation of relevant findings reported from Japan was insufficient, the Steering Committee created Working Group 1 (WG1), Dose-Rate Effects in Animal Experiments, to synthesize evidence on dose-rate effects based on animal experiments and cover research Categories 2 and 3 above.

WG1 first conducted an integrated analysis of cancer mortality data from two experiments reported by two institutes. In these studies, only female B6C3F1 mice were exposed acutely [11], and female and male B6C3F1 mice were exposed chronically [12] to ^137^Cs γ-rays. Only female mice were therefore used in the integrated analysis. WG1 showed that the DREF can be estimated based on these data using a novel statistical model that considers the change in modification by the age at exposure during chronic exposure [13]. Cumulative doses are typically used in analysis of the effect of chronic irradiation, but this implicitly assumes that exposures at different ages at exposure are of equal risk. Therefore, instead of using a simple cumulative dose, the novel statistical model uses a weighted cumulative dose, which makes it possible to take into account age differences in sensitivity.

WG1 also conducted a literature review on the dose-rate effect and relevant mechanisms in animal models, summarizing the current knowledge on biological mechanisms underlying the dose-rate effect of radiation carcinogenesis [14, 15]. The results indicate that progress has been made in studies using stem cell biology and recent state-of-the-art technologies, that some biological mechanisms of radiation effects have been identified at the cellular and molecular levels and that the results have been used to elucidate the mechanisms of these effects at the organismal level. It is expected that the results of basic studies such as these can be used to build biologically based models for estimating the risk of low-dose and low-dose-rate radiation exposure in humans, complementing findings from epidemiological studies [14, 15].

### International activity

After the ICRP issued its 2007 recommendations, an international movement toward strategic low-dose radiation research began with the European Commission’s establishment of the High Level Expert Group in 2008 and publication of its report in 2009 [16], which in turn led to the establishment of the Multidisciplinary European Low Dose Initiative (MELODI) in 2009. MELODI has continuing discussions on the development and update of their strategy and issued their latest strategic research agenda in 2022 [17]. The UNSCEAR 2020/2021 Report also provided an updated review on the mechanisms of low-dose radiation risk, with prospects for future research [18]. The Organization for Economic Cooperation and Development/Nuclear Energy Agency (OECD/NEA) organized a Scoping Meeting on Global Coordination of Low-Dose Research in 2018, and the Committee on Radiological Protection and Public Health formally launched a High-Level Group on Low-Dose Research (HLG-LDR) in 2019 to address the issue of scientific uncertainty in the health effects of low-dose and low-dose-rate radiation [19, 20]. In the USA, the Electric Power Research Institute launched the International Dose Effect Alliance in 2016, and the National Academy of Sciences, Engineering and Medicine (NASEM) published a report on research strategies related to the health effects of low-dose radiation in 2022 in response to a request from Congress [21].

During this time, PLANET members reported their activities at ICRP Symposia in 2017 and 2019 and participated in the Scoping Meeting on Global Coordination of Low-Dose Research of the OECD/NEA as an observer in 2018. PLANET’s activities were introduced at the Plenary Meeting of the HLG-LDR in 2022 and at International Dose Effect Alliance meetings in 2017, 2018, 2019, 2020 and 2022 [22]. In October 2022, PLANET held its own meeting, The International Mini Workshop on Low-Dose and Low-Dose-Rate Radiation Research: Current Status and Future Prospects, to report a summary of its latest activities to the international community. PLANET was recognized as a hub of Japanese experts in reports published by MELODI and the NASEM [17, 21].

### PLANET review of low-dose radiation research in Japan, 2017–2023

In March 2023, the PLANET Steering Committee summarized research updates in Japan over the preceding 6 years (April 2017–March 2023) based on the above-outlined five research categories [23]. Their findings are summarized by research category below.

#### Epidemiological studies of low-dose and low-dose-rate radiation appropriately designed for risk estimation (Category 1)

The Radiation Effects Research Foundation (RERF), a USA–Japan cooperative research institute, reported the dose response of solid cancer incidence and mortality adjusted for smoking [24, 25], with further reports on site-specific cancer incidence, such as esophagus and stomach, colon and rectum, liver and pancreas, lung, breast, uterine corpus and cervix, urinary tract, bladder, and kidney, prostate, central nervous system and ovary [26–35]. Radiation health effects important for radiation protection include non-cancer diseases in addition to carcinogenesis. An increased risk of non-cancer diseases including cardiovascular, respiratory and gastrointestinal diseases has been observed in A-bomb survivors, although further investigation is needed to determine if this is a causal relationship [36]. The dose response of non-cancer effects at low-dose and low-dose-rate should be investigated in the future.

The Radiation Effects Association is a public interest incorporated foundation in Japan, which conducts radiation dose registration for nuclear workers and J-EPISODE using the exposure data. They reported a large reduction in excess relative risk after adjusting for smoking, indicating that the risk of low-dose radiation, if any, is smaller than that of smoking [37–42]. The NEWS study on health effects in emergency workers for the Fukushima accident has continued with the collaboration of multiple institutions, including the National Institute of Occupational Safety and Health, Japan; Osaka University; the Japan Atomic Energy Agency, etc. [43, 44].

#### Mechanistic elucidation of low-dose and low-dose-rate radiation effects for risk estimation (Category 2)

Various studies have analyzed the response of somatic stem cells, which are possibly involved in radiation-induced diseases, to radiation and have revealed the response of somatic stem cells. Different types of epithelial cells in the rat mammary gland display distinct radiosensitivity [45–47]. Proliferation of hepatocytes continues after high-dose radiation exposure in young mice and may be related to the high susceptibility to liver cancer at this age [48]. Intestinal epithelial cells exposed to radiation are outcompeted in organoid culture in the presence of unexposed cells [49]. Radiation-induced cell loss is more prominent in the large intestine than the small intestine because of differences in the radiosensitivity of their somatic stem cells [50].

Another set of studies searched for biomarkers that represent a causal event leading to a given outcome [51] to capture the early stages of radiation-induced diseases. These include the interstitial loss of heterozygosity and/or interstitial deletions in tumors of a variety of rodent models [52–57]. Specifically, in perinatally irradiated *Ptch1*^+/−^ mice, brain tumors having interstitial loss of heterozygosity arise even after acute irradiation at 50 mGy but not after chronic irradiation at 100 mGy or 1 mGy/h [52, 53]. Interstitial deletion of *Tsc2* is specific to radiogenic kidney tumors in *Tsc2*^*Eker*/+^ rats [54]. Interstitial deletion of *Apc* is a radiation-specific mutation in intestinal tumors of *Apc*^*Min*/+^ mice [55]. In radiation-induced rat breast cancer, most mutations are common to sporadic ones, but some are only in radiogenic tumors [56, 57]. Epigenetic [58] and protein phosphorylation [59] alterations have also been reported. Phosphorylation of extracellular-regulated kinase protein is elevated in radiation-induced but not sporadic mouse lung cancer [59]. A summary report was published on an investigation of the effects and mechanisms of continuous low-dose-rate irradiation experiments at the Institute for Environmental Sciences (IES). Investigation of life span, tumor incidence, antitumor immunity, body weight, chromosomal aberrations, genetic mutations, mRNA and protein level changes, as well as transgenerational effects in continuous low-dose-rate irradiation experiments in mice [60]. Continuous irradiation (~1 mGy/h) of embryonic mice for the entire gestational period results in a slight decrease in germ cell number at 18 days of fetal age but not 10 weeks of age [61, 62], and the continuous irradiation accelerates age-associated blood pressure decline in female mice [63]. Radiation-induced structural and functional changes in the aorta that precede cardiovascular disease in mice (vascular damage, inflammation, fibrosis and hardening of the vessel wall) are dependent on the fractionation and dose rate of radiation [64–66]. In human fibroblasts, chronic irradiation (60 mGy/h) induces reactive oxygen species accumulation via a decrease in mitophagy, in conjunction with decreased expression of a group of mitophagy-related factors [67]. In human vascular endothelial cells, chronic irradiation (60 mGy/h) increases micronuclei, with a decrease in factors involved in micronucleus regulation. More than 30 other factors involved in oxidative stress and inflammation were specifically increased by chronic irradiation [68]. These results indicate cryptic consequences of radiation exposure that may later lead to observable health effects and offer the possibility of using these consequences as biomarkers for assessing low-dose and low-dose-rate radiation effects. On the other hand, dose rates below 1 mGy/h have even smaller effects that are therefore more difficult to detect and less well studied, and yet dose rates of around 1 mGy/h are considerably higher than those experienced by the public and workers. Continuous γ irradiation (0.05 mGy/day for 125–700 days, with total cumulative doses of 6.25–35 mGy) significantly reduces the frequency of translocations and dicentric chromosomes in splenic lymphocytes of mice compared to nonirradiated controls, contrary to previous reports of increased chromosome aberrations at high dose rates of 1 and 20 mGy/day [69].

It is important to conduct studies of biological effects at dose rates relevant to actual radiation protection. Compared to the dose rate for external exposure in general for radiation workers, the dose rate for nonuniform internal exposure of tissues and cells can be locally high in some cases, and it would be important to further investigate the dose–effect relationship in the future.

#### Integration of biological data for interpretation of epidemiological studies (Category 3)

Mathematical models have been developed and used to analyze animal radiobiological and epidemiological data. A statistical model was developed that considers changes in effect modification by age at exposure during chronic exposure and demonstrated its use for animal data from our institutions in estimating DREF [13]. A mathematical model was developed that simulates the consequence of cell competition, assuming different adaptation between cells exposed to radiation and intact cells, the latter of which are supposed to be predominant among cell populations at low-doses and low-dose-rates [70]. Radiation carcinogenesis is presented as an early onset of age-related cancer in a population rather than an increase in the fraction of individuals destined to develop cancer. Mathematical models were also utilized to evaluate the early onset effect of age-related cancer development by radiation [71–75]. The decrease in radiation-induced solid cancer by caloric restriction is presented as an increase in the number of stages in a multistage carcinogenesis model [76]. Acquisition of additional biological data in humans is important for future integration of biology and epidemiology. One such study analyzed DNA double-strand breaks and chromosomal aberrations in human peripheral blood cells exposed to low-dose radiation in computed tomography [77].

#### Association of age, sex, genetic predisposition and lifestyle with radiation risk (Category 4)

A number of animal experiments have elucidated various effect modifiers including age at exposure and attained age. Susceptibility to radiation-induced medulloblastoma in *Ptch1*^+/−^ mice is high in the perinatal period [52]. Susceptibility to radiation-induced breast cancer is high before puberty at low dose rates, contrary to the peak susceptibility around puberty at high dose rates [78]. Susceptibility to radiation-induced lymphoma (T and B cell) is high at a young age [79, 80]. Susceptibility to radiation-induced intestinal tumors in *Apc*^*Min*/+^ mice is high in the juvenile period [81]. Subjectively, the risk of radiation-induced lung cancer increases with age of exposure, as shown in a study of thoracic irradiation in rats [82]. Excess relative risk and excess absolute risk of breast cancer incidence decrease and increase, respectively, with age in rats [83]. Parity reduces the risk of breast cancer related to prepubertal but not postpubertal radiation exposure [83, 84]. As for genetic factors, radiation-induced breast cancer is dependent on rat strain [85, 86], and *Brca1* haploinsufficiency renders rats susceptible to breast cancer induced by prepubertal radiation exposure [87]. Diet is also an important modifier. Caloric restriction reduces the incidence of radiation-induced intestinal tumor of *Apc^Min/+^* mice and alters the mechanism of T cell lymphoma development without changing the incidence [88, 89]. Caloric restriction reduces the lifespan-shortening effect of continuous low-dose-rate irradiation in mice [90]. A high-fat diet accelerates the timing of radiation-induced breast cancer development in rats; radiation and a high fat diet act supra-multiplicatively on breast cancer incidence in rats [83, 91]. Radiation effects are also modified by positive and negative stresses. Environmental enrichment (i.e. instruments that stimulate inherent animal behaviors) promotes radiation-induced apoptosis of stem/progenitor cells in intestine, improves responses to radiation-induced DNA damage and basal immunity and suppresses chronic inflammatory responses [92, 93]. Sociopsychological stress promotes acute death of mice after radiation exposure [94]. Some studies used a mathematical approach to analyze the interaction of radiation and other factors on carcinogenesis [83, 86].

#### Data collection and database compilation (archiving), including negative data (Category 5)

Major Japanese institutions are compiling data and sample archives related to radiation epidemiology, radiation biology and radioecology. The Biosample Research Centre Database of RERF collects and stores blood, urine and lymphocytes from medical examinations of atomic bomb survivors as well as data on the samples [95, 96]. Atomic Bomb Survivors Tumor Tissue Bank of Nagasaki University stores paraffin blocks and fresh-frozen tissue samples of atomic bomb survivors [97–99]. Slide Specimen Database of Atomic Bomb Survivors of Hiroshima University provides slide specimen images of atomic bomb survivors who died shortly after the bombings, along with medical records and information on exposure status [100]. The Japan-Storehouse of Animal Radiobiology Experiments archives data and biosamples from animal experiments [101]. Environmental Radioactivity Research Network Center Sample Archive Database of Tsukuba University collects and stores environmental samples (soil, seawater, etc.), biological samples of wildlife (boar, mouse, etc.) and related data [102].

## STRATEGIC PERSPECTIVE AND FUTURE PLANS FOR PLANET

### Updating research priorities in Japan

It is a goal of PLANET to review research topics and directions on an ongoing basis to reflect progress in our understanding of low-dose and low-dose-rate radiation risk as well as the availability of new technologies. In March 2023, PLANET established the following four updated priority categories for Japanese radiation research, taking into account both recent international trends in relevant research areas and the above-listed exposure concerns in Japan (i.e. the Fukushima accident and occupational/medical exposures).

(1) Characterization of low-dose and low-dose-rate radiation risks
(2) Factors to be considered for individualization of radiation risk
(3) Biological mechanisms of low-dose and low-dose-rate radiation effects
(4) Integration of epidemiology and biology through mathematical modeling

Specific research questions are summarized by research category below, together with the expected duration of research, including short term (2–4 years), medium term (5–7 years) and long term (8–10 years).

### Characterization of low-dose and low-dose-rate radiation risks (category 1)

#### Relationship between dose-rate effects observed at the cellular, chromosomal and molecular levels and those at the organismal level (short/medium term)

It has been observed at various biological levels that lowering the dose rate without changing the cumulative dose often reduces the biological effects of radiation (i.e. the dose-rate effect). The most relevant effect to radiological protection is the risk of disease at the organismal level, which should result from underlying effects at the molecular and cellular levels. Thus, it is important to clarify how dose-rate effects at the organismal level relate to those at the molecular and cellular levels. It is also important to conduct studies to determine biological effects at even lower dose rates that are relevant to actual radiological protection.

#### Biological effects of spatially heterogeneous radiation energy deposition in cell populations (short/medium term)

Very low-dose and low-dose-rate radiation results in exposure that is heterogeneous in both time and space. In considering the biological effects of radiation, it is necessary to take into account the spatial heterogeneity of energy deposition within a cell population. Thus, it is important to investigate the potential singularity related to this heterogeneity. ICRU Report 86 considers an approach that, in terms of microdosimetry, better clarifies the nature of energy deposition in discrete targets than when using absorbed doses. It recommends the use of particle radiance, which contains information on particle type and time, and its use allows dosimetry due to non-uniform irradiation [103]. The ICRU report98 emphasizes the improvement of current radiation bioeffect models through rigorous description of experimental conditions and analysis of microsimulation results [104]. These approach methods will reveal the detailed aspects of the initial deposition of energy on biological targets due to low-dose and low-dose-rate irradiation. The results will contribute to the basis of assessing health effects of low-dose and low-dose-rate radiation exposures and internal exposures from unevenly distributed radionuclides such as insoluble particles.

#### Effects of internal exposures and their biological mechanisms (short/medium term)

Internal exposures include ingestion of tritium and inhalation of insoluble particles. Discharge of tritium has been a central issue for the Fukushima accident as well as nuclear fuel reprocessing and nuclear fusion. The impact of organically bound tritium should be studied using state-of-the-art techniques. Inhalation of insoluble radioactive cesium particles also attracted attention in the wake of the Fukushima accident. The effect of these particles has not been tested scientifically. Comparison of the effects of exposure during various stages of gametogenesis and at fertilization may be of special note. For example, in internal exposure experiments in mice, local irradiation with Auger electrons [105–107] reduced spermatogenesis. It has also been reported that internal contamination with radionuclides emitting α particles, Auger electrons and γ rays induces abnormalities in sperm heads [108].

#### Risk expression considering the uncertainties in its estimation (short/medium term)

Individualization of risk assessment will require the development of new risk indicators in which populations are grouped according to risk, or new ways of providing risk information from a wider perspective. Individualization (including the use of biomarkers) and understanding of risk information are particularly relevant to medical exposure. Although the nominal risk coefficients are more or less averaged, this topic addresses the question of what approaches are appropriate for incorporating individualized risk information into radiological protection.

#### Transgenerational effects in humans and animals (medium term)

Transgenerational effects have been observed in some strains of mice exposed to high-dose-rate radiation [109], but have not necessarily been confirmed either independently or mechanistically. Recent technologies such as deep sequencing have enabled quantitative examination of transgenerational effects [110, 111]. Sequencing studies of trios—parents who survived an atomic bombing and their children—have been conducted [112] and more are planned [113].

Advances in molecular genetics have made it possible to reveal animal pedigrees in detail, allowing for rigorous evaluation of de novo mutation rates. In a study on canines living around Chornobyl, the genomes of wild canines from high-dose and low-dose areas were examined over 15 generations, and differences were found in their pedigrees [114]. However, it is unclear whether this is related to low-dose-rate radiation exposure, which needs to be investigated in the future. It is also an issue for future study whether this technology can be applied to humans and compared with results in animals. Animal studies may offer advantages for analyzing the dose and dose-rate dependence of genomic changes and their underlying mechanisms.

#### Archiving of data and samples related to low-dose and low-dose-rate radiation effects (medium/long term)

Experimental, epidemiological and radioecological data, as well as samples are stored by various institutions, but these have a risk of loss for various reasons, including financial limitations and retirement of individuals in charge. As these resources are extremely valuable, measures should be taken to ensure their effective and sustainable use in cooperation with relevant domestic and international institutions.

#### Epidemiology of radiation workers with adjustments for potential lifestyle-related confounders (long term)

Epidemiological studies of radiation workers require proper adjustments for potential confounders, including lifestyle factors such as smoking, alcohol consumption, diet and exercise. The availability of such information in the J-EPISODE cohort offers a unique opportunity for assessing the health effects of low-dose-rate radiation exposure relevant to the workplace.

#### Epidemiology of medical exposures related to advanced technologies (long term)

Epidemiological studies should target patients exposed to radiation from advanced technologies in oncology and nuclear medicine, including carbon ion radiotherapy with various irradiation techniques (e.g. passive scattering, active scanning and rotational gantries) [115]. In the radiopharmaceutical therapy field, a number of α- and β-emitting radionuclides (^131^I, ^212^Pb, ^225^Ac, ^90^Y, ^117^Lu, ^223^Ra, ^227^Th, etc.) are clinically available or in development [116], and ^211^At has recently been added [117]. ICRU Report 96 [116] defines regions at risk as critical tissues that may suffer significant morbidity or loss of function if irradiated. In particular, regions of the body that may be at increased risk of stochastic effects, such as solid cancers and leukemias, are also given a new term, Regions at Risk for Secondary Effects (RAR_SE_). RAR_SE_ may include the whole body. The related dosimetry might be necessary for epidemiology.

#### Radiation effects and radiation risk assessment in space (long term)

Japan will continue to contribute to international collaborations on manned space explorations, where protection of astronauts from space radiation is an issue. The USA National Aeronautics and Space Administration leads the relevant research, and ICRP Task Group 115 is making an effort to summarize the results and make new recommendations for future directions of study [118]. Warranted study areas include the distinction of radiation effects from other stresses such as microgravity, the combination of these effects and the relevance of the complex radiation quality therein.

### Factors to be considered for individualization of low-dose-rate radiation risk (Category 2)

#### Lifestyle factors (short/medium term)

Relevant lifestyle factors include, but are not limited to, diet, smoking and exercise. Currently, available information about such factors is limited to modification of the effect of acute high-dose-rate exposures. These factors are closely related to immune and metabolic functions, which may also be affected by radiation. Understanding their involvement may provide suggestions for methods to reduce risk, such as lifestyle changes and chemical interventions.

#### Genetic factors (short/medium term)

Genetic factors can influence individual radiosensitivity. The advent of *in vivo* genome editing technologies has enabled the creation of new genetically modified animal models that mimic rare germline mutations in humans. Relevant new models will be useful for studying radiation health effects and dose-rate effects in an individualized manner.

#### Gender and the reproductive endocrine system (short/medium term)

Consideration of the impact of sex and individual differences in the reproductive endocrine system (e.g. age at puberty, hormone levels) is increasingly important for understanding radiation health effects. In addition, the studies suggest that dose rates as low as 1 mGy/day can affect reproductive organs [60], which may influence carcinogenesis in many other organs via changes in hormone levels and other mechanisms.

#### Age at exposure (medium term)

Age at exposure has been shown to impact the health effects of acute radiation exposure differently in different organs. Presumably, such organ-specific effects involve different underlying mechanisms of radiosensitivity. It may be more challenging to clarify whether and how such modifications differ at low dose rates because radiosensitivity changes as a function of cumulative dose. Animal studies coupled with statistical techniques will likely play an important role here.

### Biological mechanisms of low-dose and low-dose-rate radiation effects (Category 3)

#### Exploration of mechanism-based biomarkers for risk estimation (short/medium term)

It is important to have sensitive biomarkers that reflect the molecular consequences of and cellular responses to low-dose and low-dose-rate radiation to investigate the health effects of such exposures. The use of biological samples from irradiated humans, animals and tissue culture models will enable the search for biomarkers with causal relationships to health effects. Comprehensive analyses by genomic, transcriptomic, proteomic, metabolomic and metagenomic approaches will serve for this exploration.

#### Mechanisms of dose-rate effects related to tissue stem cell kinetics (short/medium term)

Tissue stem cells are considered to be an origin of cancer and other age-related diseases. It is therefore necessary to examine the contribution of radiation-induced changes to stem cell kinetics, including those that occur via microenvironmental changes, in the health effects of low-dose-rate radiation. Possible mechanisms may include genetic and epigenetic alterations, clonal evolution, senescence, inflammation and stem cell competition.

#### Role of epigenetic mechanisms at low-doses and low-dose-rates (medium/long term)

Because DNA damage is less likely to occur at lower dose rates, mechanisms that do not involve DNA damage, such as epigenetics, may play a more important role in radiation-related risk. Epigenetics is associated with aging and therefore related to both cancer and non-cancer diseases, although its significance in the biological effects of radiation is not well established. It is therefore necessary to explore the role of epigenetics in the effects of low-dose and low-dose-rate radiation.

#### Impact of organ-to-organ crosstalk on radiation risk (medium/long term)

Tissues and organs make up a network that acts through various systems including the endocrine, nervous and immune systems. Epithelial cells and the microenvironment are also interrelated. Such connections are relevant to radiation-induced phenomena such as inflammation, aging and abscopal effects. It is currently unclear how such crosstalk is relevant to radiation health risks such as cancer and non-cancer diseases or to their dose-rate effects. Further exploration may lead to a new paradigm of radiation exposure risk.

#### Integration of molecular/cellular/organismal and animal/human knowledge through a parallelogram approach (long term)

The parallelogram approach refers to a method of predicting adverse effects of an agent in humans by extrapolating the axis of ‘molecular/cellular events leading to organismal consequences’ in animals to the parallel axis in humans [51]. It is vital to examine how this approach could be used for radiation effect research.

### Integration of epidemiology and biology through mathematical modeling (Category 4)

#### Quantitative characterization of radiation carcinogenesis based on parallel analyses of animal and epidemiological data (short/medium term)

It is possible to reevaluate the mechanism of radiation carcinogenesis by comparing the fits of conventional excess relative risk and excess absolute risk models as well as other novel models (e.g. earlier onset models [71, 72, 75]). Applying an identical mathematical model to animal and epidemiological data will benefit the identification of species specificity and cross-species similarity, and further the examination of whether those approaches can be used in humans for risk transfer between populations as well as animal-to-human risk transfer.

#### Development of dose–response models that consider the mechanisms of biological effects of low-dose and low-dose-rate radiation (medium/long term)

The analysis of animal and epidemiological data using biologically based mathematical models is important to better understand the dose response of radiation health effects. This may involve the development of adverse outcome pathways, which are an evidenced-based consolidation of causal relationships between multiple molecular and biological events that end in an adverse outcome of an agent, with the quantitative nature of those relationships being considered. Based on such models, a wide range of dose rates could be categorized in terms of risk estimation taking into account the dose-rate dependence of the outcome and the underlying biological mechanisms that operate depending on the dose rate.

## NEW PLANET WGS

**Fig. 2:**
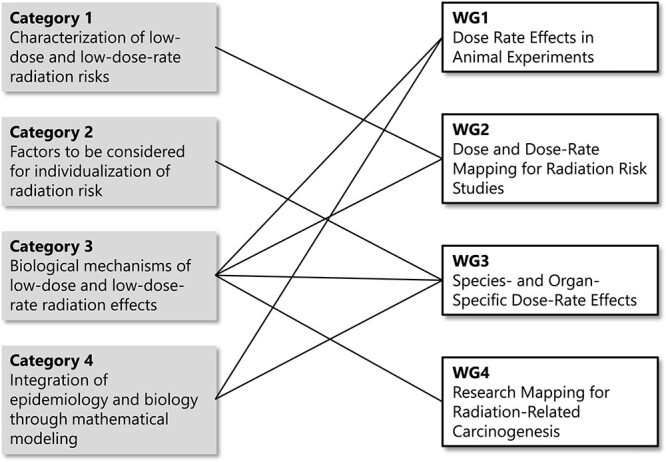
Relationship between research categories and WGs.

In response to the updated research priority categories, PLANET set up three new WGs as detailed below. The relationship between the new research categories and WGs is illustrated in Fig. 2.

###  

#### WG2: Dose and dose-rate mapping for radiation risk studies

WG2 aims to review the dose and dose-rate ranges that have observable effects in epidemiological and radiobiological research relevant to risk estimation and to categorize those doses and dose rates in terms of the mechanisms underlying dose-rate effects. The tasks of WG2 are:

(1) To identify the range of doses and dose rates at which evidence is available on observable effects at the molecular, cellular and organismal levels, including early responses, site-specific cancers, non-cancer diseases and transgenerational effects.

(2) To characterize the biological context of those observed dose-rate effects according to dose and dose-rate ranges, discuss relevant research needs and publish the results as a review article.

WG2 relates to research priority Categories 1 (characterization of low-dose and low-dose-rate radiation risks) and 3 (biological mechanisms of low-dose and low-dose-rate radiation effects).

#### WG3: Species- and organ-specific dose-rate effects

Recent advances in stem cell dynamics (e.g. cell death and cell cycle) and differences observed between animal species and organs may be related to differences in radiation cancer risk among different organs and species, and further to the integration of animal studies and epidemiology. WG3 aims to review and organize relevant studies. The tasks of WG3 are:

(1) To summarize findings on radiation carcinogenesis in terms of the organ at risk, their dose and dose-rate dependence, as well as relevant modifying factors.

(2) To summarize the similarities and differences in the above findings among animal and human studies.

(3) To discuss possible mechanisms underlying the dose-rate effect of carcinogenesis in those species and organs and publish the results as a review article.

WG3 relates to research priority Categories 2 (factors to be considered for individualization of radiation risk), 3 (biological mechanisms of low-dose and low-dose-rate radiation effects) and 4 (integration of epidemiology and biology through mathematical modeling).

#### WG4: Research mapping for radiation-related carcinogenesis

WG4 aims to map major findings regarding the process of low-dose-rate radiation effects with the aid of adverse outcome pathway–based methods to identify area(s) where further research is needed. The tasks of WG4 are:

(1) To list the possible processes related to carcinogenesis (including relevant non-cancer effects) at low-dose-rates based on published evidence.

(2) To clarify the singularities and characteristics of the processes relevant to low-dose-rates by comparing the findings with those at high-dose-rates.

(3) To identify unexplored areas and pinpoint studies needed to understand the whole process of radiation carcinogenesis at low-dose-rates.

(4) To discuss the extrapolation of the above findings to cancer risks of low-dose-rate radiation in humans and publish the results as a review article.

WG4 relates to research priority Category 3 (biological mechanisms of low-dose and low-dose-rate radiation effects). The current paradigm of radiation carcinogenesis is based on the mutational hypothesis, which forms the basis of the linear-no-threshold theory. On the other hand, available evidence suggests the possibility of non-mutational processes (e.g. tumor promotion where radiation-induced microenvironmental changes affect spontaneously initiated cells), necessitating consolidation of relevant findings.

## CLOSING REMARKS

The ICRP plans to update its 2007 recommendations in the early 2030s. To contribute to this effort, researchers should focus on specific objectives that can inform the ICRP discussions. PLANET has proposed a list of specific research topics that should be prioritized in Japan and has launched three WGs to explore selected issues. In the coming years, PLANET will lead the research and discussions on these prioritized areas among research institutes, universities and relevant academic societies in Japan that are involved in low-dose radiation research. In addition, PLANET will continue to share information and collaborate with international consortia that can actively contribute to protecting people from the biological effects of low-dose and low-dose-rate radiation.
